# Quantitative Field Testing *Heterodera glycines* from Metagenomic DNA Samples Isolated Directly from Soil under Agronomic Production

**DOI:** 10.1371/journal.pone.0089887

**Published:** 2014-02-24

**Authors:** Yan Li, Gary W. Lawrence, Shien Lu, Clarissa Balbalian, Vincent P. Klink

**Affiliations:** 1 Department of Biochemistry, Molecular Biology, Entomology and Plant Pathology, Mississippi State University, Mississippi State, Mississippi, United States of America; 2 Department of Biological Sciences, Mississippi State University, Mississippi State, Mississippi, United States of America; Dowling College, United States of America

## Abstract

A quantitative PCR procedure targeting the *Heterodera glycines* ortholog of the *Caenorhabditis elegans uncoordinated-78* gene was developed. The procedure estimated the quantity of *H. glycines* from metagenomic DNA samples isolated directly from field soil under agronomic production. The estimation of *H. glycines* quantity was determined in soil samples having other soil dwelling plant parasitic nematodes including *Hoplolaimus*, predatory nematodes including *Mononchus*, free-living nematodes and biomass. The methodology provides a framework for molecular diagnostics of nematodes from metagenomic DNA isolated directly from field soil.

## Introduction

Plant parasitic nematodes present a major problem for the cultivation of *Glycine max* (soybean) [1]–[5]. Among them, the dominant problem is the soybean cyst nematode, *Heterdera glycines* [2]–[5], [6]. *H. glycines* was first detected in North Carolina in 1954 [7] and subsequently spread rapidly to as far away as Mississippi by 1957 [8]. The host range of *H. glycines* is broad, updated to over 399 plant species [9]–[13]. *H. glycines* are a bisexual, cyst-forming species consisting of six life stages including the egg, four juvenile stages (J1–J4), and the adult stage [6], [14], [15]. The duration of the *H. glycines* life cycle is 3–4 weeks, but this time frame may be influenced by environmental conditions [15]. *H. glycines* feed from their plant host by producing a nurse cell called a syncytium which forms by the fusion and incorporation of approximately 200 cells [16]. Depending upon the environment, several generations of *H. glycines* can be completed in a typical soybean growing season. Adding to management problems, eggs within cysts can remain dormant for 9 years [17]. Management is further complicated because cultivars under production, while experiencing as much as a 30% decrease in yield loss, may appear symptom-free [18]. Infection of soybean by *H. glycines* results in annual losses of $ 1 billion in yield in the U.S. [19] and about $15 billion worldwide. To mitigate these losses, management of *H. glycines* is accomplished partly by the use of soybean genotypes that are resistant to infection [20]–[24]. Although the use of resistant genotypes is a component of recommended control measures [25], consistent use of soybean cultivars with the same sources of resistance can lead to adaptation of the existing *H. glycines* population to cultivars with that source of resistance [26]. Along with the use of resistant germplasm, the application of crop rotation and nematicides are also used practices in the management of *H. glycines* [27].

A long-used agricultural management strategy is to determine the types and quantities of nematodes present in the soil before planting and this has been particularly important for managing soybean cultivation in *H. glycines-*infested fields [13], [28]–[31]. While these types of tests are traditionally done by trained nematologists, the analyses can be time consuming (i.e. up to 100 days or longer). Therefore, molecular diagnostic tests which could be done in a shorter time frame (i.e. hours) would be useful in the management of many plant-parasitic nematodes [32]–[39]. Quantitative PCR (qPCR) is a procedure that specifically assays the PCR reaction by incorporating a molecular tag whose amplification products are measured during the PCR reaction. The qPCR method works quantitatively in pathogen detection and measurement because it detects the pathogen by the using the amount of DNA present in a sample to obtain a cycle threshold (Ct) value which corresponds to the level of infection [40]. The qPCR methodology has been shown to be a very powerful tool in pathogen detection, used to identify and quantify various bacteria, fungi, and viruses [41]–[44]. Population estimates for nematode species such as *M. javanica*, *Pratylenchus zeae*, and *Xiphinema elongatum* have been determined with qPCR on native soil samples from trial plots [45]. The qPCR procedure has also been used to determine the quantity of the plant parasitic nematode *Rotylenchulus reniformis* from metagenomic DNA isolated directly from field plots [46].

It is hypothesized that conserved gene sequences can be used in determining the number of *H. glycines* from metagenomic DNA isolated directly from soil. In the analysis presented here, highly conserved *H. glycines* homologs of the *C. elegans* uncoordinated gene family genes [47] were screened for their use as biomarkers against other phylogenetically related and unrelated nematodes [48]. The molecular diagnostic was able to specifically identify *H. glycines* from metagenomic DNA samples isolated directly from soil under agricultural production. Furthermore, a single, *H. glycines* could be identified from the metagenomic DNA sample. The methodology is rapid, and can be performed *en masse* in a single afternoon.

## Results

### Probe Identification

**Figure 1:**
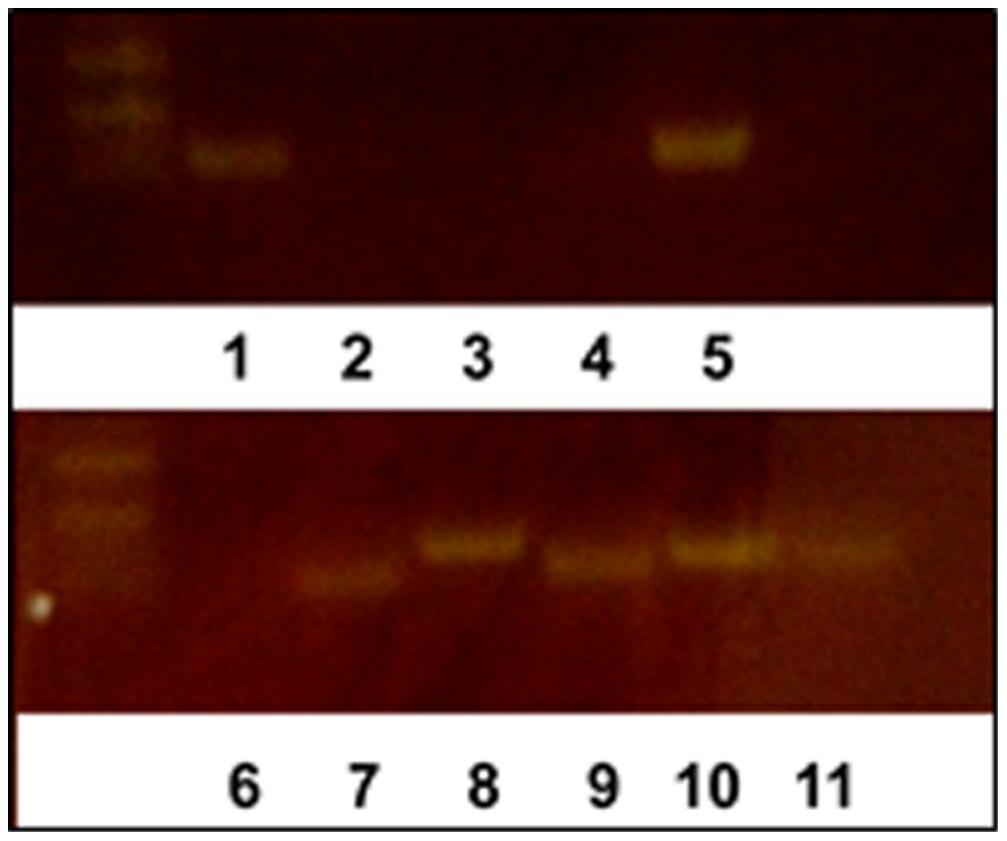
Screening of Hg-unc primer pairs. Lane 1, Hg-unc 9; Lane 2, Hg-unc 22; Lane 3, Hg-unc 31; Lane 4, Hg-unc 52; Lane 5, Hg-unc 115; Lane 6, Hg-unc 101; Lane 7, Hg-dys-1; Lane 8, Hg-nep-1; Lane 9, Hg-unc 89; Lane 10, Hg-unc 78; Lane 11, Hg-MRCK-1.

**Figure 2:**
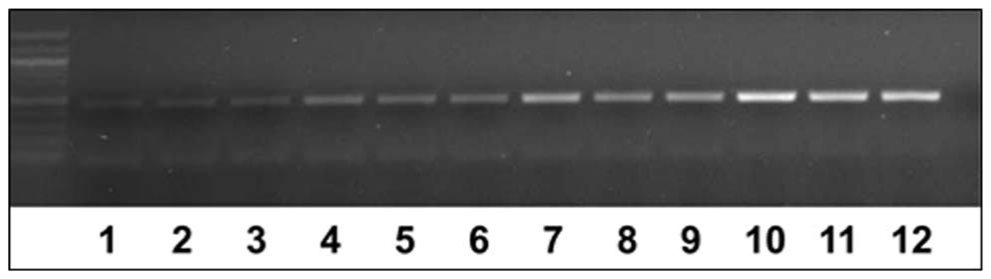
DNA amplification of Hg-unc-78 from different numbers of pure, extracted *H. glycines* (SCN) J2 cultured in soil in greenhouse. Lanes 1–3, 1 SCN; Lanes 4–6, 10 SCN; Lanes 7–9, 100 SCN; Lanes 10–12, 1,000 SCN.

Alkharouf et al. [49] analyzed 8,334 *H. glycines* unigenes that resulted in the identification of numerous genes involved in essential aspects of their biology. From those sequences, 11 genes were targeted for their use as molecular probes to determine the level of soil infestation by *H. glycines* (**Table 1**). The 11 primer pairs, designed from these gene sequences, were used in a series of PCR reactions to identify genes that would robustly amplify *H. glycines* DNA isolated from pure single cysts as compared to a control gene (**Figure 1**). As demonstrated by an increase in the amount of amplification product, semiquantitative PCR tests demonstrated Hg-unc-78 primer pairs were able to detect increasing amounts of DNA from increasing numbers of *H. glycines* J2 (**Figure 2**). Gene sequencing confirmed the PCR amplicon was Hg-unc-78 (**data not presented**).

**Table 1:** PCR primer pairs for *H. glycines* homologs of *uncoordinated* (*unc*) class genes.

*H.glycines*gene	*H.glycines*est	ProductSize	Primer	TM
Hg-unc-9	CB281382	124	F-CCATGGTGCGCTATTTGTCA	62.95
			R-CTGCCCCCAAATTGTTTGAA	63
Hg-unc-9	CB281382	253	F-GCGCGAAAGGACGATGATTTTTC	66.5
			R-CTTACGGCCGGACGAATACCTCTC	66.6
Hg-unc-22	CB378705	120	F-GACGAAATTGTGGCCGAGTC	62.91
			R-AAATTGTCCCGCGTCCTCTT	63.11
Hg-unc-31	CB378080	110	F-CTCCGATGGTTGTCCGCTAC	62.91
			R-GGTTGAGCAACCGTCTTTGC	63.05
Hg-unc-52	CK350534	114	F-ACCGCAGGTGTACGATGGTT	62.69
			R-CCGTAGGCGGTCACTTTGTC	62.92
Hg-unc-78	CB238521	97	F-CGTTTTTGGGACACCACACA	62.79
			R-TGCTGTCCTCAGACCACGAA	63.04
		qPCRprobe	GAAGTCGGAGTTCGCTCTTCTTTCG	70.98
Hg-unc-78	CB238521	313	F-GTGGAGACCAATCGGGCAAAATC	66.4
			R-GAAAGGAGGGCCTTCGAAAATGG	66.5
Hg-unc-89	CB379143	120	F-GCGCGGTACTGACGAAAGTC	63.19
			R-GCAGGACAGTTTCCGCATTC	63.04
Hg-unc-89	CB379143	289	F-CCCGTACACACATTTCCGCAGTC	66.3
			R-CAGCCGACCATCGAGTTCCATAC	66.5
Hg-unc-101	CB379764	94	F-CATGCAAGGCAACAGATTCG	62.7
			R-TAACAGCGCACATCCAAACG	63.09
Hg-unc-115	CK350435	103	F-ACGGAAGTCGCGCTATTCAA	63.06
			R-GTCGTTGTCCACGGAAGAGG	63.01
Hg-dys-1	CB934909	124	F-GCTATTTGCCGGTCGAACAA	63.28
			R-TTGTCCAATCTCGCGGCTAT	62.92
Hg-dys-1	CB934909	303	F-GTTTCCGATCGTTGGACTTCG	66.5
			R-GCTGGTGCATTGCCTCTGTTTC	66.4
Hg-nep-1	CB824545	114	F-TATTCGGGCGTCAAAAATGC	63.04
			R-GCCAATCACTGCTCCAATCC	62.9
Hg-MRCK-1(control)	CB380016	125	F-CCACCGACACGTCCAACTTT	63.27
			R-GAAGGTGAAGCCGATGAACG	63.02

### Detecting *H. glycines* using Metagenomic DNA Isolated Directly from Soil

**Figure 3:**
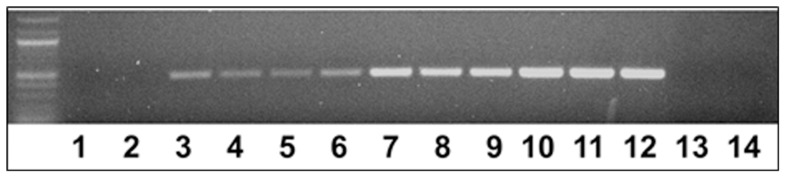
DNA amplification of Hg-unc-78 from metagenomic DNA isolated directly from soil containing different numbers of *H. glycines* (SCN). Lanes 1–3, 1 SCN; Lanes 4–6, 10 SCN; Lanes 7–9, 100 SCN; Lanes 10–12, 1,000 SCN; Lane 13, without primer (control); Lane 14, without DNA (control).

**Figure 4:**
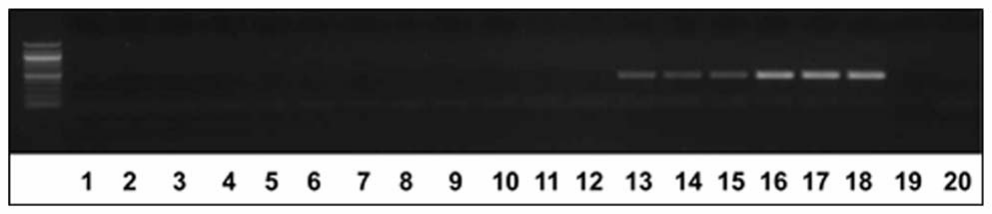
The specificity of the Hg-unc78 PCR reaction from DNA isolated from different numbers of pure, extracted off-target nematodes as compared to DNA isolated from pure, extracted *H. glycines*. Lanes 1–3; 100 Rr; Lanes 4–6, 1,000 Rr; Lanes 7–9, 100 RKN; Lanes 10–12, 1,000 RKN; Lanes 13–15, 100 SCN; Lanes 16–18, 1,000 SCN; Lane 19, without primers (control); Lane 20, without DNA (control). Rr, *Rotylenchulus reniformis*; RKN, *Meloidogyne incognita*.

**Figure 5:**
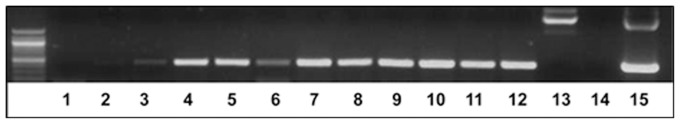
DNA amplification of the Hg-unc78 from metagenomic DNA samples. Lanes 1–3, metagenomic DNA including 1 SCN J2; Lanes 4–6, metagenomic DNA including 10 SCN J2; Lanes 7–9, metagenomic DNA including 100 SCN J2; Lanes 10–12, metagenomic DNA including 1,000 SCN J2; Lane 13, cloned gene without primers (control); Lane 14, without DNA (control); Lane 15, positive control, amplification product is Hg-unc 78 amplified from the cloned gene.

The experiments then focused on determining if *H. glycines* DNA could be isolated and detected directly from soil samples. Metagenomic DNA isolation, followed by PCR, demonstrated that the Hg-unc 78 amplification product could be detected as a band in samples with as few as 1 added J2 (**Figure 3**). To further confirm the specificity of Hg-unc 78 primers, experiments on agriculturally significant off-target plant-parasitic nematodes including *R. reniformis* [50] (Hoplolaimidae) and *M. incognita* (root knot nematodes, RKN) (Heteroderidae), along with *H. glycines*, DNA were used in PCR experiments. No amplification was achieved in reactions containing *R. reniformis* or *M. incognita* DNA, but amplification was successful on reactions containing *H. glycines* DNA (**Figure 4**). This outcome indicates that the primers designed to amplify Hg-unc 78 is specific to *H. glycines*. Since it was possible to amplify target *H. glycines* DNA from soil containing as little as a single *H. glycines* J2 nematode, experiments were done whereby metagenomic DNA was isolated directly from field samples containing numerous parasitic and free-living nematodes as well as other living organisms. Soil samples were collected in triplicate and a visual assessment of the nematodes present within the soil sample were determined (**Table 2**). This procedure determined that the soil contained other species of nematodes that are more distantly related to *H. glycines*, including *Hoplolaimus* (sp.) [51] (Hoplolaimidae) and *Mononchus* (sp.) [52] (Mononchidae). Known numbers of *H. glycines* were then added to the soil samples and the metagenomic DNA was isolated directly from the soil as described previously. The amplification profile of the Hg-unc-78 DNA is shown (**Figure 5**). The demonstration of increasing amounts of amplification product in samples having increasing amount of added *H. glycines* indicated that it would be possible to adapt qPCR to the detection method.

**Table 2:** Quantification of major nematode fauna from field soil.

Microplots	*Hoplolaimus* spp	*Mononchus* spp	Free living
**1**	1287.5	772.5	772.5
**2**	772.5	2317.5	257.5
**3**	257.5	0	515
**4**	257.5	257.5	515

### qPCR Estimate of *H. glycines*

**Figure 6:**
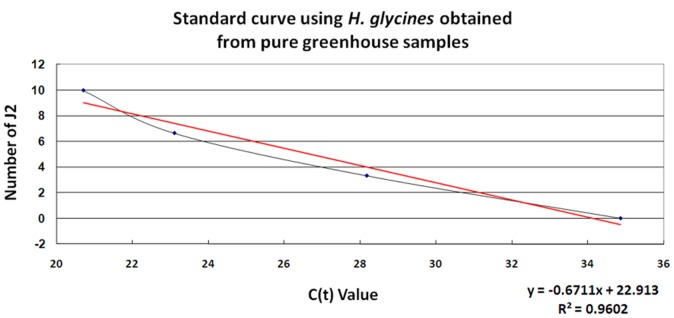
Estimation of *H. glycines* population size by qPCR. Standard curve obtained using pure greenhouse samples, (b) soil samples. The statistical significance between the actual number of SCN and the estimated number are provided.

**Figure 7:**
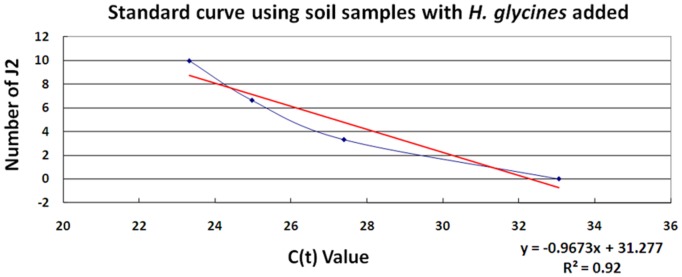
Estimation of *H. glycines* population size by qPCR. Standard curve obtained from metagenomic DNA isolated directly from soil samples. The statistical significance between the actual number of SCN and the estimated number are provided.

The qPCR experiments began with pure, greenhouse cultured *H. glycines* J2s (**Figure 6**). From this standard curve, it is demonstrated that the Hg-unc-78 qPCR primers are able to measure, quantitatively, the different amount of *H. glycines* DNA within the samples. This measurement allows for the correlation of the quantity of the amplified DNA amount to estimate number of *H. glycines* J2s. The same procedure then was used to estimate the number of *H. glycines* J2s directly from metagenomic DNA isolated directly from soil samples (**Figure 7**). Subsequently, actual soil samples under agricultural production were assayed using qPCR for *H. glycines* (**Table 3**). Metagenomic DNA was isolated from those samples and used in qPCR experiments. Since the number of *H. glycines* is a pool of both cysts and J2s, it was necessary to estimate the number of J2s per cyst. Using that estimate, a correlation between the counted *H. glycines* as compared to the qPCR estimate is provided (**Table 3**). DNA sequencing of the qPCR product demonstrated the specificity of the reaction by revealing the amplification product was Hg-unc-78 (**data not presented**).

**Table 3:** qPCR estimation of *H. glycines* from field soil samples.

Sample	J2/pint	Cysts/pint	Cyst to J2*	Actual NO. J2/pint	qPCR J2 estimate/pint	fold difference
1	0	8	1,600	1,600	8,394.38921	0.19
2	0	16	3,200	3,200	5,599.6761	0.57
3	0	24	4,800	4,800	7,692.002288	0.62
4	24	16	3,200	3,224	4,928.816234	0.65
5	0	16	3,200	3,200	2,369.447	1.35
6	63	24	4,800	4,863	1,857.50756	2.62
7	8	8	1,600	1,608	421.9439991	3.81
8	0	8	1,600	1,600	370.9181429	4.32
9	16	47	9,400	9,416	1,557.579433	6.05
10	32	32	6,400	6,432	747.7392119	8.62

## Discussion

The determination of the numbers of specific types of plant parasitic nematodes in the soil is important in agriculture because it allows for understanding threshold populations that could detrimentally affect plant productivity. This knowledge, in turn, will allow growers to make informed choices in terms of cultivar and/or crop selection as nematode populations can vary from year to year. The analysis presented here resulted in the development of a simple and reliable molecular diagnostic technique that determines the number of *H. glycines* from metagenomic DNA isolated directly from field soil under agricultural production. During the course of the analysis, gene sequences for *H. glycines* homologs of *C. elegans unc* genes were identified, tested and analyzed for their reliability in molecular diagnostics. During the analysis, qPCR primers were designed and used on metagenomic DNA isolated directly from actual soil samples from real agricultural sites. While the focus of the analysis was low numbers of *H. glycines* (1–1,000 individual J2s), it is expected that the methodology would also be reliable for analyzing field samples that are more highly infested.

### Hg-unc-78 as a Molecular Probe

Gene sequences analyzed in Alkharouf et al. [49] was mined for genes that could be used as reliable molecular probes for the presence of *H. glycines* in actual field samples under agricultural production. Genes that function in nematode movement, an important aspect of parasitism, and were also conserved in their primary structure to *C. elegans* were identified and used for PCR primer design. Many of these genes were *unc* genes, first described in *C. elegans* [53]. The experiments presented here show that it is possible to use highly conserved DNA sequences that are homologous to *unc* genes from *H. glycines* and use them to specifically amplify gene fragments from as few as 1 J2. The specificity was shown when attempts in amplification reactions of off-target nematodes and, in particular, metagenomic DNA isolated directly from soil samples. DNA sequence analyses of the DNA amplification products from the PCR reactions show that the amplification product perfectly matches its target DNA from *H. glycines*. Lastly, using the same primers to amplify target DNA from metagenomic DNA isolated directly from soil samples reveals that it is possible to amplify the target as shown by sequencing reactions of that amplification product to as low as a single *H. glycines* J2. Furthermore, in regard to the major nematode species found in Mississippi soils, the procedure appears to distinguish *H. glycines*. However, all nematodes have not been examined.

### Quantitative PCR

The qPCR experiments were designed with the goal of determining the numbers of *H. glycines* in soil samples collected from actual field sites under agricultural production. From the described PCR studies, it was determined that it would be possible to identify a single *H. glycines* from metagenomic DNA. Similar observations have been made for *R. reniformis* [46]. The DNA samples isolated from pure cultures of *H. glycines* could then be used in the development of a standard curve from which it was possible to estimate the number of *H. glycines*. With this capability, it was then tested whether it would be possible to take metagenomic DNA samples isolated from soil having predefined numbers of *H. glycines* to quantify the number of *H. glycines*. As shown, the Hg-unc-78 primer set could be used in experiments to accurately estimate the number of J2s in the sample. The quantitative analysis, using qPCR, demonstrates that low numbers of *H. glycines* can be identified reliably from the metagenomic samples. Notably, the quantity of *H. glycines* has been determined through visual analysis from actual soil samples under agricultural production, serving as experimental samples. Metagenomic DNA then was isolated from those samples and used for subsequent qPCR analyses. The analysis demonstrated that the qPCR did not perfectly determine the exact number of *H. glycines* in the agricultural samples. The same observation was made for *R. reniformis* [46]. However, it is well known that *H. glycines* are not evenly distributed within soil samples [27]. Thus, the limits of detection may have been met. Further experimentation is required to explore this problem further.

A key question remaining is whether different compositions of soil will affect the DNA isolation efficiency. In Mississippi where the experiments were carried out, there are 5 major soil types. The soil types vary among alfisols, entisols, inceptisols, ultisols and vertisols [54]. It is possible that these different soil compositions could affect the efficiency of the DNA isolation and amplification. While no major influence was observed on amplification from the different sample types used in the various analyses, a thorough analysis on different soil types from the obtained unknown samples requires further testing.

## Materials and Methods

### Ethics Statement

Since the focal point of the research was plant parasitic nematodes, IACUC regulations were not relevant to this study.

### Materials Statement

No specific permits were required for the described field studies because the soil samples were collected on the grounds owned by Mississippi State University and contained no endangered or protected species as determined by a stereomicroscopic analysis of soil.

### Plant Inoculation

Prior to nematode inoculation, *G. max* seedlings are grown in a sterilized sand: soil mix (1∶1) in clay pots for a period of 7 to 14 days. The nematodes of second stage juveniles were counted, concentrated and diluted to a final concentration of 2,000 J2 per 3 ml which were then added to each root. This meant that 3 ml of inoculum that contained 2,000 nematodes were added to each root system on each plant by inoculating on two spots evenly.

### Plant and Nematode Culture

The target nematode *H. glycines* and off-target *M. incognita* (root knot nematode, RKN) [55]–[56] (Heteroderidae) and *R. reniformis* (reniform nematode) [48] (Hoplolaimidae) were cultured under ambient conditions in a greenhouse at the Mississippi Agriculture and Forestry Experiment Station (MAFES), RR Foil Plant Science Research Center, North Farm, Mississippi State University. Supplemental fluorescent light was provided to bring the day length to a 16 hour day/8 hour night cycle. Temperatures were kept in a constant temperature range between 28.9–34.4°C. Nematodes were cultured in 500 cm^3^ diameter clay pots for a period of 2–6 months in a 50∶50 mixture of a fine sandy loam (46.25% sand, 46.50% silt, and 7.25% clay) and sand (93.00% sand, 5.75% silt, and 1.25% clay). Harvesting cysts was accomplished by massaging the infected roots in water. Massaging was achieved by placing the infected root mass between the index finger and thumb. The index finger and thumb were gently rubbed together with the root mass between them. This gentle rubbing activity dislodged the cysts so that they could be collected on a 150 µm pore sieve under a constant water steam. The cysts are not damaged because of their protective hardened nature. Cysts were collected by rinsing them through nested 850 µm pore sieve (debris) and a 150 µm sieve (cyst). Then cysts were rinsed out of the 150 µm pore sieve into a beaker with 100 ml of water. Juveniles were collected by rinsing them through 250 µm pore sieve (debris) and 45 µm pore sieve (J2) and transferred to a beaker with 100 ml water in it for counting. Cysts were crushed by using Janke & Kunkel IKA-WERK crushing machine (IKA-WORKS, INC), for 30 seconds to 1 minute with a timer. Removal of debris smaller than the eggs was done by washing the slurry though a 25 µm pore sieve. The eggs were transferred to a beaker with 100 ml water in it for counting [57].

### Soybean Cyst Nematode DNA Isolation

To isolate DNA from a single cyst, a single cyst was picked up under a stereomicroscope and placed into a 1.5 ml micro-centrifuge tube, with 14 µl sterile distilled water. The tube was placed in −20°C for 1 hour to freeze the cyst (or liquid nitrogen for 1 min). A glass rod sterilized with 75% ethanol was used to grind the cyst containing mixture until it is thawed. A 3 µl 10×PCR buffer and 3 µl proteinase K was then added. The material was incubated at −20°C for at least 2 hours. The solution was incubated for 90 min at 65°C to denature the protein, and 15 min at 9°C to unwind the DNA. The solution was centrifuged for 1 min at 8,000 rpm/h with the supernatant transferred to a new 1.5 ml microcentrifuge tube. The DNA was stored at −20°C for later PCR analysis.

### Gene Screening

*H. glycines* genes identified in Alkharouf et al. [49] were used for primer design according to Showmaker et al. [46] who used the identical methodology for quantitative molecular detection of *Rotylenchulus reniformis* DNA from metagenomic samples isolated from soil. Highly conserved genes from Group 1 [49], having 266 genes with *E*-values between 0 and 1*E*-100, were the focus of the analysis because no other *Heterodera* spp. agricultural important species has been published to be found in Mississippi soils at this time. Many of these genes included the *uncoordinated* (*unc*) class were first described in *C. elegans*. The genetically-defined *unc* genes group contains 114 different members and function in muscle focal adhesion, architecture and stimulation [53].

### Metagenomic DNA Isolation

Metagenomic DNA from soil isolations were conducted by using the Powersoil DNA extraction kit (MO BIO Laboratories, Inc; Carlsbad, CA) using the manufacturer’s protocol with modifications. The modifications included using 0.3 grams of soil instead of 0.25 grams of soil in step for developing the quantitative PCR standard curve and adding 0.1 ml of nematode suspension (water containing the numbers of J2 extracted from greenhouse cultured soil samples). In its place, 0.1 ml of the nematode suspension, extracted from either greenhouse pots or field soil, was pipetted into the bead beating tube. Secondly, in the step when first instructed to remove the supernatant, a standard volume of 550 ml of supernatant was removed from each tube. This ensured for downstream applications. The DNA was eluted from the spin column in 50 µl of nuclease free water (Promega; Madison, WI) and stored at –20°C.

### PCR Reaction Conditions

For PCR, a 50 µl PCR reaction consisting of 2 µl DNA template, 5.0 µl 10×PCR buffer, 1.0 µl dNTP, 3.0 µl MgCl_2_, 0.25 µl recombinant Taq Polymerase, 1.25 µl of 100 nM forward and reverse primers each, 36. µl nuclease free water (Ambion) (Promega) was used. The reaction conditions, as reported by Agudelo et al. [39] were modified to include a 2 minute pre-denaturation step at 94°C. The procedure then followed the Agudelo et al. [39] protocol that included a denaturation at 94°C for 45 sec, annealing at 59°C for 45 sec and primer extension at 72°C for 60 sec for 40 cycles. The PCR reaction products were run out by gel electrophoresis on a 1% agarose gel with 0.01% SYBR-Green incorporated into the gel. The DNA amplification products were visualized and recorded with digital imagery using a FOTO/Analyst Apprentice System (FOTODYNE Inc.; Hartland, WI).

### Cloning and Sequencing

After using Accuprime polymerase for the PCR, the DNA amplification products were extracted on the gel by using the gel extraction kit (QIAQuick Gel Extraction Kit), mixing 4 µl of the gel purified DNA with 1 µl of salt solution and 1 µl of TOPO vector for the TOPO cloning reaction (pENTR Directional TOPO Cloning Kits, Invitrogen) in a 1.5 ml microcentrifuge tube. The tube was incubated at room temperature for 5 min and then placed on ice. Chemically competent *E. coli* stored at −80°C was thawed on ice. Transformation began by distributing the *E. coli* cells in a separate 1.5 ml microcentrifuge tube, at a volume of 20 µl each, which is proceeded on ice. Then, 3 µl of TOPO cloning reaction was added to the *E. coli* cells, tapping the tube gently. The *E. coli* solution were incubated on ice for 15 min followed by heat shocking the *E. coli* cells at 42°C for 30 seconds on the heat block. The *E. coli* solution then was placed back on ice immediately. Subsequently, 100 µl of SOC media was added to the tube and shaken at 37°C for 1 hour. After incubation, 70 µl of the solution were plated out on LB-kanamycin plates. The plates were incubated at 37°C overnight for 12–24 hours. The next day, single colonies were picked and placed in a 50 ml centrifuge tube containing LB-kanamycin culture agar. The tubes were cultured for 37°C for no more than 16 hours, usually around 15 hours. A plasmid prep was done on the culture (QIAPrep, Miniprep, QIAGEN).

To confirm that the DNA amplification in both PCR and qPCR reactions were products of *H. glycines* DNA and not spurious amplification of off-target DNA, DNA amplification products were run out electrophoretically on and then isolated from the 1% agarose gels. The DNA was purified using the Qiaquick Gel Extraction Kit (Qiagen; Valencia, CA) as according to the manufacturer’s specifications. The isolated DNA was ligated into the pGEM-T Vector System II (Promega). The ligation reaction was shuttled into competent JM109 *E. coli* cells and selected on 50 µg/ml ampicillin on LB-agar plates. Colonies were selected and grown in liquid culture in LB media containing 50 µg/ml ampicillin. Plasmid DNA was isolated from the bacteria using the Qiaprep kit (Qiagen). The DNA from the plasmid preps was sequenced to determine if the DNA amplification product was correctly amplifying the proper target. The DNA sequence was trimmed using the Crimson Editing freeware (http://www.crimsoneditor.com/). In this procedure, the pGEM-T Vector DNA sequence was trimmed leaving the qPCR-generated sequence. The trimmed sequence was blasted in GenBank using the blastn query option. This additional quality control step demonstrated the accuracy of the qPCR reaction conditions.

### Quantitative PCR

Taqman 6-carboxyfluorescein (6-FAM) qPCR probes (MWG Operon; Birmingham, AL) were used. The 6-FAM probes have a maximum excitation at 495 nm and maximum emission at 520 nm. The qPCR quencher was the Black Hole Quencher (BHQ1) (MWG Operon), with maximum excitation at 534 nm. Assays were conducted for primers that produced a single amplicon and had no off target amplification. The qPCR reaction conditions included a 20 µl Taqman Gene Expression Master Mix (Applied Biosystems; Foster City, CA), 0.9 µl 100 µM forward primer, 0.9 µl 100 µM reverse primer, 2 µl 2.5 µM 6-FAM (MWG Operon) probe and 4.4 µl metagenomic template DNA. The conditions were a denaturation at 94°C for 45 sec, annealing at 54°C for 45 sec and primer extension at 72°C for 60 sec for 40 cycles. The qPCR reactions were performed on an ABI 7300 (Applied Biosystems).

To generate a standard curve for the amount of *H. glycines* in a soil sample, estimates of approximately 1,000 nematodes in 0.1 ml of water were placed into the Powersoil DNA isolation kit bead beating tubes and extracted as described previously, replicated this by 2 more times. A 1∶10 serial dilution series of DNA extracted from approximately 1,000 nematodes was created and used for generation of the standard curve by qPCR. To evaluate the accuracy of the standard curve, samples containing 0, 1, 10 and 100 *H. glycines* nematodes were generated by carefully hand collecting them under a stereoscope, 3 replicates of each, and isolated the DNA by the Powersoil DNA isolation kit as described previously. The qPCR methodology works quantitatively because it detects pathogens by using the amount of DNA present in a sample to obtain a cycle threshold (C(t)) value which corresponds to the amount of target DNA (Livak and Schmittgen, 2001). The lower the C(t), the greater the amount of the corresponding DNA (target organism) is present in a sample.

### *H. glycines* Estimation

50 g of soil was weighed and put into a cylinder. Subsequently, 50 ml of water was put in the cylinder. Because of the volume of the soil, the total volume increased to 70 ml, meaning the 50 g of soil was equal to a volume of 20 ml. With 50 g taking up 20 ml, 0.3 g of soil takes (0.3 g×20 ml)/50 g = 0.12 ml. So the estimated number of J2 in 1 pint = 473.176 ml×C(t)/0.12.
